# Effective nonlinear responses of three-phase magnetoelectric composites

**DOI:** 10.1038/s41598-022-19143-2

**Published:** 2022-09-06

**Authors:** Chien-hong Lin, Fang-Yu Liu

**Affiliations:** Department of Mechanical Engineering, National Cheng Kung University, 1 University Road, Tainan City, 70101 Taiwan

**Keywords:** Composites, Computational methods

## Abstract

A computational method, dubbed simplified unit-cell micromechanics model, is generalized and applied to establish the effective nonlinear responses of three-phase magnetoelectric composites that are composed of two distinct magnetostrictive and piezoelectric phases embedded in elastic polymer matrices. The nature of nonlinear constitutive behavior of each constituent is expected to significantly influence the overall responses of the composites. To obtain the effective nonlinear responses, a mathematical linearization is first introduced to perform the constitutive linearization for the nonlinear materials, and the resulting constitutive equations are then unified and nested into the micromechanics model followed by iterations in order to minimize errors from the linearization process. For the purpose of comparison, we also reformulate the well-established Mori–Tanaka micromechanics model insofar as its mathematical structure is aligned with that of the simplified unit-cell model. Numerical results are first validated against limited experimental measurements available in literature. Parametric studies are then conducted in order to reveal the effect of phase constitutive laws, volume fractions, and geometries on the overall nonlinear responses of there-phase magnetoelectric composites. The contributions of this work complement those of earlier studies that prevalently devoted to two-phase magnetoelectric composites and linear magneto-electro-elastic coupled responses only.

## Introduction

Magnetoelectric materials, that induce an electric polarization by an applied magnetic field or vice versa generate a magnetization via an external electric field, has been shown to hold the significant technological promise of a large impact on a number of applications, especially on novel multifunctional devices. The magnetoelectric coupling in a single-phase crystal is relatively weak for practical use. Examples of such compounds are Cr_2_O_3_, Gd_2_CuO_4_, Sm_2_CuO_4_, etc.^1^. Therefore, research quickly began to progress to investigate how piezomagnetic (or magnetostrictive) materials can be combined with piezoelectric (or electrostrictive) substances to create two-phase magnetoelectric composites which typically exhibit giant magnetoelectric effects and are ready for practical applications. In such composites, the magnetoelectric coupling is an indirect coupling, via mechanical strain, between two active solids that individually show magneto-elastic and electro-elastic couplings, respectively. Such magnetoelectric property is known as product property^2^ of composite materials, and can be tailored by appropriate choice of phases with piezomagnetism (or magnetostriction) and piezoelectricity (or electrostriction) and their connectivity^3^. Consequently, there is a growing body of literature that presents corresponding mathematical models for modeling of magnetoelectric composites.

Early works are by Harshé^4^, Harshé et al.^5^, and Avellaneda and Harshé^6^ who studied composites combining piezomagnetic CoFe_2_O_4_ and piezoelectric BaTiO_3_ constituents and proposed a cubes model to predict the overall magnetoelectric coefficient of the composites with 0–3 and 2–2 connectivities. Nan^7^ presented a theoretical framework based on a Green's function method and perturbation theory to predict magnetoelectric behavior in two-phase CoFe_2_O_4_/BaTiO_3_ composites having 1–3 and 0–3 connectivities. The Mori–Tanaka theory is employed to study CoFe_2_O_4_/BaTiO_3_ magnetoelectric composites with 0–3, 1–3 or 2–2 connectivities by several researchers: Huang and Kuo^8^, Li and Dunn^9^, Koutsawa^10^, and Wang et al.^11^, for example. Similarly, the generalized self-consistent scheme is used by Tong et al.^12^ for modeling 1–3 CoFe_2_O_4_/BaTiO_3_ composites. Aboudi^13^ proposed an asymptotic homogenization method to estimate the effective moduli of 1–3 and 2–2 composites containing CoFe_2_O_4_ and BaTiO_3_ phases. Kim^14^ utilized an exact matrix method while Koutsawa^15^ formulated a method based on the mechanics of structure genome for simulating CoFe_2_O_4_/BaTiO_3_ composites having 2–2 and 1–3 connectivities, respectively. All aforementioned papers are focused on the magnetoelectric composites composed of two distinct CoFe_2_O_4_ and BaTiO_3_ phases whose constitutive equations are assumed as a linear relationship between field variables.

When active constituents, such as magnetostrictive Terfenol-D, piezoelectric PZT or PVDF, are employed to form a magnetoelectric composite, the overall constitutive behavior of the composite exhibits significantly nonlinear response due to the material nonlinearity of each individual phase. For example, Crawley and Anderson^16^ had experimentally shown that a significantly nonlinear strain response is observed when a piezoelectric PZT-G1195 plate subjected a large electric driving field. Similarly, Jiles and Thoelke^17^ had experimentally investigated a Terfenol-D rod undergoing an applied magnetic field. The resulting piezomagnetic strain and magnetic flux density are nonlinear as well. Several mathematical models focusing on the overall nonlinear behavior of magnetoelectric composites, as a result, are developed. For instance, Jin and Aboudi^18^ considered a magnetoelectric composite with nonlinear Terfenol-D reinforcements in a linear PVDF matrix, and implemented the high-fidelity generalized method of cells method to estimate the overall nonlinear magnetoelectric responses for 1–3, 0–3 and 2–2 Terfenol-D/PVDF composites. Hu et al.^19^ investigated the nonlinear magnetoelectric response of a tri-layered Terfenol-D/PZT/Terfenol-D composite. In their paper, they considered that both Terfenol-D layer and PZT layer show significantly nonlinear behavior and developed a two-level micromechanics model to obtain the overall coupled response of the tri-layered composite. To this end, most theoretical approaches that have been forwarded in the context of magnetoelectric composites are directed toward two-phase composites only.

However, the monolithic Terfenol-D, PZT, and BaTiO_3_ etc. are not easy to be molded into desired shapes for producing devices; they are brittle and would be susceptible to failure even only withstand small strains. Increased attention, thus, is soon being directed toward how magneto-elastic and electro-elastic materials can be combined with polymer matrices to create three-phase magnetoelectric composites. Efforts in this area are frequently concerned with developing mathematical models with linearly coupled magnetic, electrical, mechanical and thermal properties via a homogenization approach. For example, Lee et al.^20^ employed the Mori–Tanaka theory and finite element method to estimate the effective properties of three-phase magneto-electro-elastic composites having CoFe_2_O_4_ and BaTiO_3_ fibers embedded in polymer matrices. Later, Tang and Yu^21^ studied the same three-phase composites as Lee et al.^20^ did, but they included thermal coupling effect in their modeling scheme, i.e., the variational asymptotic method for unit cell homogenization.

Nonetheless, the efforts in theoretical understanding of the effective nonlinear response of three-phase magnetoelectric composites is quite limited with respect to that of the considering nonlinear constitutive behavior in each composite phase. A recent article by Zhang et al.^22^, they theoretically studied the magnetoelectric effect of a three-phase layered magnetoelectric composite composed of Terfenol-D, PZT-8H and an inactive FeCuNbSiB layer with high magnetic conductivity. The phase constitutive law employed by Zhang et al.^22^ is nonlinear only for magnetostrictive constituent but is linear for piezoelectric material. Furthermore, their work concerned merely about 2–2 connectivity. As mentioned by Nan et al.^23^, the most common connectivity types of magnetoelectric composites covers 1–3, 0–3, and 2–2 types. When the responses of three-phase magnetoelectric composites with embedded magnetostrictive, piezoelectric, polymer constituents are sought, theoretical approaches that consider various connectivities and nonlinear constitutive behaviors have to be included. Therefore, a true need still exists for a robust model that establishes explicit macroscopic constitutive equations and the associated local field distributions for three-phase magnetoelectric composites. The presentation of such a framework is the focus of this study.

The purpose of this work is to present two micromechanics models to reveal the effective nonlinear responses of three-phase magnetoelectric composites; that is the effective constitutive behavior of the composites will be modeled. The approach of the first-moment secant linearization is first applied to linearize the nonlinear constitutive laws of magneto-elastic and electro-elastic bulk media, respectively, and the resulting constitutive equations are then unified with linear elastic constitutive relation of an elastic matrix. The final constitutive equation is linearized, unified, field-dependent, and nonlinear coupled for magneto-electro-elastic heterogeneous media, and later to couple it with micromechanics predictions, the simplified unit-cell and the Mori–Tanaka schemes respectively, in order to obtain the expressions for the effective field-dependent moduli and effective nonlinear responses of three-phase magnetoelectric composites. Simulation results are first validated against experimental data available in literature. Numerical results are then presented for typical composite connectivities, 1–3, 0–3 and 2–2, which illustrate the interesting behavior of three-phase magnetoelectric composites, particularity emphasizing on the existence of magnetoelectricity in a composite constructed by three nonmagnetoelectric materials.

## Basic equations of nonlinear magneto-electro-elasticity

A three-phase composite concerned in this study is constituted by piezoelectric, magnetostrictive, and polymeric phases. Herein, we make a simplifying assumption, for analytical purposes, that the composite is under small deformations and within the scopes of electrostatics and magnetostatics (i.e., we disregard the transient response of a composite). The constitutive relations of these three phases are summarized here, respectively. First, for piezoelectric phase, this study is restricted in a polarized piezoelectric material subject to a large driving electric field but without exceeding the coercive field. In such case, it is reasonable to assume that the effect of depolarization and polarization switching are negligible during the operation for actuation or sensing. As a result, the nonlinear constitutive laws derived by Tiersten^24^ is utilized in this study as shown below

1 $$ \sigma_{ij} = C_{ijkl}^{{}} \varepsilon_{kl} - e_{kij}^{{}} E_{k} - \frac{1}{2}b_{klih}^{{}} E_{l} E_{k} , $$

2 $$ D_{i} = e_{ikl}^{{}} \varepsilon_{kl} + \kappa_{ij}^{{}} E_{j} + \frac{1}{2}\chi_{ijk}^{{}} E_{k} E_{j} , $$

where the field variables are stress $\sigma_{ij}$, strain $\varepsilon_{ij}$, electric displacement $D_{i}$, and electric field $E_{i}$. The material properties are the elastic stiffness $C_{ijkl}^{{}}$, the third-order and fourth-order piezoelectric stress constants $e_{ijk}^{{}}$ and $b_{ijkl}^{{}}$ respectively, and the second-order and third-order permittivities $\kappa_{ij}^{{}}$ and $\chi_{ijk}^{{}}$ respectively. Second, for magnetostrictive phase, this study, similarly, assumes a magnetostrictive material subject to a large driving magnetic field but without exceeding the coercive field. Under the circumstances, it is reasonable to presume that the effect of magnetization/demagnetization and domain reorientations are negligible during the operation for actuation or sensing. Consequently, the nonlinear constitutive laws proposed by Carman and Mitrovic^25^ is employed here given as

3 $$ \sigma_{ij} = C_{ijkl}^{{}} \varepsilon_{kl} - q_{kij}^{{}} H_{k} - \frac{1}{2}x_{klih}^{{}} H_{l} H_{k} , $$

4 $$ B_{i} = q_{ikl}^{{}} \varepsilon_{kl} + \mu_{ij}^{{}} H_{j} + \frac{1}{2}\omega_{ijk}^{{}} H_{k} H_{j} , $$

where $B_{i}$ and $H_{i}$ are magnetic flux density and magnetic field, respectively. $q_{ijk}^{{}}$ and $x_{ijkl}^{{}}$ are the third-order and fourth-order piezomagnetic stress constants, respectively, while $\mu_{ij}^{{}}$ and $\omega_{ijk}^{{}}$ are the second-order and third-order magnetic permeabilities, respectively. Third, for polymeric phase, it is assumed as a conventional solid which is linear behavior among all elastic, electric, and magnetic fields and is uncoupled among any of them. It is noted that, although $C_{ijkl}^{{}}$ in Eqs. (1) and (3) are the same symbol, the elastic stiffness are in general different for different phases.

Here, a first-moment secant linearization is applied to linearize the nonlinear constitutive equations. For example, Eqs. (1) and (2) can be cast under a secant linearized form as:

5 $$ \sigma_{ij} = C_{ijkl}^{{}} \varepsilon_{kl} - h_{kij}^{{}} E_{k} , $$

6 $$ D_{i} = e_{ikl}^{{}} \varepsilon_{kl} + g_{ij}^{{}} E_{j} , $$

where

7 $$ h_{kij}^{{}} = e_{kij}^{{}} + \frac{1}{2}b_{klij}^{{}} E_{l} , $$

8 $$ g_{ij}^{{}} = \kappa_{ij}^{{}} + \frac{1}{2}\chi_{ijk}^{{}} E_{k} . $$

Similarly, Eqs. (3) and (4) are rewritten for linearized magneto-elastic constitutive equations accordingly:

9 $$ \sigma_{ij} = C_{ijkl}^{{}} \varepsilon_{kl} - j_{kij}^{{}} H_{k} , $$

10 $$ B_{i} = q_{ikl}^{{}} \varepsilon_{kl} + i_{ij}^{{}} H_{j} , $$

where

11 $$ j_{kij}^{{}} = q_{kij}^{{}} + \frac{1}{2}x_{klih}^{{}} H_{l} , $$

12 $$ i_{ij}^{{}} = \mu_{ij}^{{}} + \frac{1}{2}\omega_{ijk}^{{}} H_{k} . $$

With the linearized constitutive equations, Eqs. (5), (6), (9) and (10), in hand, a set of constitutive equations that directs the nonlinear interaction of magnetic, electric and elastic fields in a magnetoelectric medium can be derived through a mathematical superposition of those equations that share common strain and stress field variables. Subsequently, magnetoelectric coupling tensors $a_{ij}^{{}}$ and $\lambda_{ij}^{{}}$ are instinctively derived from the resultant equations, given as

13 $$ \sigma_{ij} = C_{ijkl}^{{}} \varepsilon_{kl} - h_{kij}^{{}} E_{k} - j_{kij}^{{}} H_{k} , $$

14 $$ D_{i} = e_{ikl}^{{}} \varepsilon_{kl} + g_{ij}^{{}} E_{j} + a_{ik}^{{}} H_{k} , $$

15 $$ B_{i} = q_{ikl}^{{}} \varepsilon_{kl} + \lambda_{ik}^{{}} E_{k} , + i_{ij}^{{}} H_{j} . $$

Magnetoelectric coefficients are caused by mechanically coupled magnetostrictive and piezoelectric phases in a three-phase composite: it is present in neither phases individually.

In addition, the kinematic equations that relate elastic displacement $u_{i}$, electric potential $\phi_{i}$, and magnetic potential $\varphi_{i}$ to strain, electric field, and magnetic field, respectively, are given by:

16 $$ \varepsilon_{ij} = \frac{1}{2}(u_{i,j} + u_{j,i} ), $$

17 $$ E_{i} = - \phi_{,j} , $$

18 $$ H_{i} = - \varphi_{,i} , $$

where the comma notation indicates a derivative.

Moreover, the equilibrium equations that satisfy mechanical equilibrium and the conservation of magnetic and electric fluxes in a magneto-electro-elastic solid, in absence of body forces and free charges, are defined by:

19 $$ \sigma_{ij,j} = 0, $$

20 $$ D_{i,i} = 0, $$

21 $$ B_{i,i} = 0. $$

Equations (13)–(21) summarizes the governing equations of a quasi-static magneto-electro-elastic problem for a magnetoelectric composite containing nonlinear constituents.

For coupled multi-physics problems, it will be convenient in the sequel to define the vectors ${{\varvec{\Sigma}}}$ and ${\mathbf{Z}}$ as follows:

22 $$ {{\varvec{\Sigma}}} = \left\{ {\sigma_{11} ,\;\;\sigma_{22} ,\;\;\sigma_{33} ,\;\;\sigma_{23} ,\;\;\sigma_{13} ,\;\;\sigma_{12} ,\;\;D_{1} ,\;\;D_{2} ,\;\;D_{3} ,\;\;B_{1} ,\;\;B_{2} ,\;\;B_{3} } \right\}^{{\rm T}} , $$

23 $$ {\mathbf{Z}} = \left\{ {\varepsilon_{11} ,\;\;\varepsilon_{22} ,\;\;\varepsilon_{33} ,\;\;2\varepsilon_{23} ,\;\;2\varepsilon_{13} ,\;\;2\varepsilon_{12} ,\;\;E_{1} ,\;\;E_{2} ,\;\;E_{3} ,\;\;H_{1} ,\;\;H_{2} ,\;\;H_{3} } \right\}^{{\rm T}} , $$

such that a set of the constitutive equations in Eqs. (13)–(15), consequently, can be unified into a single equation by using Voigt and Nye’s contracted notations, i.e.,

24 $$ {{\varvec{\Sigma}}} = {\mathbf{L}}({\mathbf{E}},{\mathbf{H}}){\mathbf{\rm Z}}, $$

where ${\mathbf{L}}({\mathbf{E}},{\mathbf{H}})$ is a 12 × 12 matrix. It contains field-dependent (or arithmetic) material properties that are the outcomes of the linearization process. The parenthesis used in Eq. (24) explicitly indicates that the arithmetic matrix ${\mathbf{L}}$ is indeed a function of fields ${\mathbf{E}}$ and ${\mathbf{H}}$, and should not be confused with a multiplication operation. As convention, a boldface letter is used to denote a vector, matrix or tensor of any order, while a lightface letter is utilized to represent a scalar or a component of a tensor. The superscript T in Eqs. (22) and (23) stands for transpose operation. Equation (24) will be utilized throughout the subsequent micromechanics analysis that deals with the nonlinear responses of three-phase magnetoelectric composites.

## Micromechanics formulations for three-phase magnetoelectric composites

The simplified unit-cell micromechanics model that simulates three-phase magnetoelectric composites with 1–3, 0–3 and 2–2 connectivities is first formulated in this section. For the purpose of comparison, we further reformulate the Mori–Tanaka micromechanics model insofar as its mathematical structure is aligned with that of the simplified unit-cell model.

### Simplified unit-cell model

**Figure 1:**
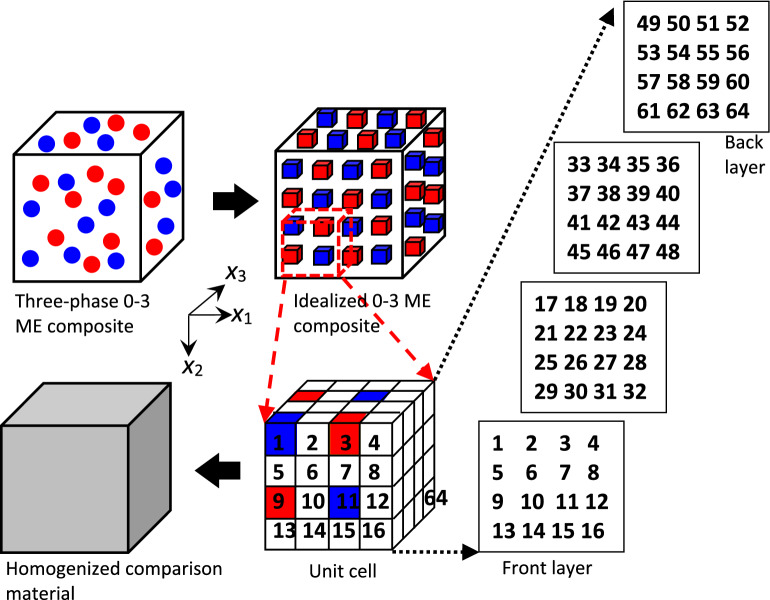
Homogenization process of the simplified unit-cell micromechanics model for a three-phase 0–3 ME composite. ME an abbreviation for magnetoelectric.

The simplified unit-cell model has been recently employed by Lin and Lin^26^ for the prediction of the nonlinear behavior of two-phase magnetostrictive-piezoelectric composites, and by Lin and Liu^27^ for the simulation of three-phase polymer matrix smart composites. Lin and Lin^28^, Zhan and Lin^29^ and Shen and Lin^30^ applied the unit-cell model for the study of two-phase magnetostrictive composites. Early works by Lin and Muliana^31–33^, Tajeddini et al.^34^, and Muliana and Lin^35^ are examples of modeling of two-phase piezoelectric composites based on the unit-cell model. Besides, the two-phase unit-cell model itself was first developed by Lin and Muliana^31^ who generalized the four-cell model that was early proposed by Haj-Ali^36^. Regarding the historical development of unit cell-based micromechanics model and its related models, we refer the reader to a recent publication of Lin^37^ for reference. Further applications of the simplified unit-cell model are on composite structures, for example, hybrid active composite structures investigated by Lin and Muliana^38^ and functionally graded piezoelectric beams studied by Lin and Muliana^39^. In these papers, extensive comparisons with experimental data or other computational approaches, i.e., finite element, Mori–Tanaka etc., have been performed in order to verify the reliability of the two-phase unit-cell model. In the present article, the simplified unit-cell model is further generalized and applied to establish the effective nonlinear responses of three-phase magnetoelectric composites. A three-phase composite microstructure is first idealized as a periodically distributed array that is constructed by elementary cubes shown in Fig. 1. A unit cell is then defined by a cubic array to the extent that it is able to generate the overall microstructure by means of repeating itself. For a three-phase composite, the simplest unit cell is therefore identified as a collection of 64 subcells. The unit cell here can also be viewed as an idealized representative volume element whose effective behavior is representative of that of a composite material as a whole. In the case of a 0–3 magnetoelectric composite, as shown in Fig. 1, the magnetostrictive and piezoelectric particles (blue and red ones) occupy the subcell 1, 3, 9, 11, 33, 35, 41 and 43 in a spatially equivalent manner, while the polymer matrix (white one) engages the rest of the subcells.

The field of each subcell is regarded as an average field; as a result, the expression of the average field of a magnetoelectric composite can be derived by volume‐weighted average over the entire unit cell, that is

25 $$ {\overline{\mathbf{Z}}} = \sum\limits_{\alpha = 1}^{64} {c^{(\alpha )} {\mathbf{Z}}^{(\alpha )} } , $$

26 $$ {\overline{\mathbf{\Sigma }}} = \sum\limits_{\alpha = 1}^{64} {c^{(\alpha )} {{\varvec{\Sigma}}}^{(\alpha )} } . $$

An overbar denotes that a quantity is in an average sense. The superscript $(\alpha )$ represents the identity of a subcell and is named as a set of consecutive integers, 1–64. $c^{(\alpha )}$ is the volume fraction of the subcell $\alpha$ and is determined by $c^{(\alpha )} = {{V^{(\alpha )} } \mathord{\left/ {\vphantom {{V^{(\alpha )} } {\sum\nolimits_{\alpha = 1}^{64} {V^{(\alpha )} } }}} \right. \kern-\nulldelimiterspace} {\sum\nolimits_{\alpha = 1}^{64} {V^{(\alpha )} } }}$, where $V^{(\alpha )}$ is the volume of the subcell $\alpha$. Depending on each constituent that occupies specific subcells, the volume fraction of each phase in a three-phase magnetoelectric composite can be calculated. The constitutive equation of a unit cell is defined as follows:

27 $$ {\overline{\mathbf{\Sigma }}} = {\mathbf{\overline{L}\overline{Z}}}, $$

where ${\overline{\mathbf{L}}}$ is regarded as the effective property of a three-phase magnetoelectric composite. The constitutive equation of a subcell is of course given by

28 $$ {{\varvec{\Sigma}}}^{(\alpha )} = {\mathbf{L}}^{(\alpha )} {\mathbf{Z}}^{(\alpha )} . $$

The volume average of the local field in the subcell $\alpha$ is related to the applied overall field by a concentration factor tensor ${\mathbf{A}}^{(\alpha )}$^40^ below

29 $$ {\mathbf{Z}}^{(\alpha )} = {\mathbf{A}}^{(\alpha )} {\overline{\mathbf{Z}}}. $$

Substituting Eq. (29) into Eq. (28) to replace ${\mathbf{Z}}^{(\alpha )}$, we obtain

30 $$ {{\varvec{\Sigma}}}^{(\alpha )} = {\mathbf{L}}^{(\alpha )} {\mathbf{A}}^{(\alpha )} {\overline{\mathbf{Z}}}. $$

Substituting for ${{\varvec{\Sigma}}}^{(\alpha )}$ from Eq. (30) into Eq. (26), we arrive at the expression for the effective response:

31 $$ {\overline{\mathbf{\Sigma }}} = \sum\limits_{\alpha = 1}^{64} {c^{(\alpha )} {\mathbf{L}}^{(\alpha )} {\mathbf{A}}^{(\alpha )} } {\overline{\mathbf{Z}}}. $$

Comparing Eq. (27) with Eq. (31), we arrive at the result for the effective property:

32 $$ {\overline{\mathbf{L}}} = \sum\limits_{\alpha = 1}^{64} {c^{(\alpha )} {\mathbf{L}}^{(\alpha )} {\mathbf{A}}^{(\alpha )} } . $$

From Eqs. (31) and (32), it is obvious that once the concentration factor tensor ${\mathbf{A}}^{(\alpha )}$ is determined, the overall response ${\overline{\mathbf{\Sigma }}}$ and average property ${\overline{\mathbf{L}}}$ of a three-phase magnetoelectric composite can be obtained. In order to evaluate the concentration factor tensor in the simplified unit-cell model, micromechanical relations between the subcells and constitutive models for all subcells are imposed. The micromechanical relations are derived based on the continuity conditions of displacements, tractions, electric potentials, normal electric displacements, magnetic potentials, and normal magnetic flux densities at the interfaces among the subcells. For the simplified unit‐cell model having 64 subcells as shown in Fig. 1 and employing three physical domains (magnetic, electric and elastic), Eq. (29) results in 768 (64 × 12) independent variables ${\mathbf{Z}}^{(\alpha )}$. Thus, we need to define 768 equations which are obtained from the micromechanical relations as listed in Supplementary 1 that also provides additional pictograms to clearly illustrate how the three-phase model simulates 1–3 and 2–2 connectivity types. The novelty of this work is that the present model can unify three typical connectivity types, i.e., 0–3, 1–3 and 2–2 with the least number of subcells, i.e., 64 subcells, into a unit cell for modeling of three-phase magnetoelectric composites. The equations can be written in a matrix form as:

33 $$ \mathop {\left[ {\mathbf{P}} \right]}\limits_{768 \times 768} \mathop {\left\{ {{\mathbf{Z}}^{(1)} ,\;{\mathbf{Z}}^{(2)} ,\; \cdots ,\;{\mathbf{Z}}^{(64)} } \right\}^{{\rm T}} }\limits_{768 \times 1} = \mathop {\left[ {\mathbf{Q}} \right]}\limits_{768 \times 12} \mathop {\left\{ {{\overline{\mathbf{Z}}}} \right\}}\limits_{12 \times 1} . $$

From Eq. (33), the concentration factor matrix ${\mathbf{A}}^{(\alpha )}$ in linearized relations are therefore determined by

34 $$ \mathop {\left\{ {{\mathbf{A}}^{(1)} ,\;{\mathbf{A}}^{(2)} ,\; \cdots ,\;{\mathbf{A}}^{(64)} } \right\}^{{\rm T}} }\limits_{768 \times 12} = \mathop {\left[ {\mathbf{P}} \right]^{ - 1} }\limits_{768 \times 768} \mathop {\left[ {\mathbf{Q}} \right]}\limits_{768 \times 12} , $$

where the superscript −1 represents inverse operation. The linearized micromechanical relations are exactly satisfied only when all subcells exhibit linear constitutive responses; as a result, Eq. (34) directly provides the required concentration factor matrix to estimate the effective linear response of a three-phase magnetoelectric composite. In contrast, due to the nonlinear responses in the magnetostrictive and piezoelectric subcells, the linearized micromechanical relations will usually violate the constitutive equations. This inconsistency is defined by a residual written as

35 $$ \left\{ {\mathbf{R}} \right\} = \left\{ \begin{gathered} {\mathbf{Z}}^{(1)} - {\mathbf{A}}^{(1)} {\overline{\mathbf{Z}}} \hfill \\ {\mathbf{Z}}^{(2)} - {\mathbf{A}}^{(2)} {\overline{\mathbf{Z}}} \hfill \\ \quad \quad \vdots \hfill \\ {\mathbf{Z}}^{(64)} - {\mathbf{A}}^{(64)} {\overline{\mathbf{Z}}} \hfill \\ \end{gathered} \right\}. $$

Numerical iterations using computers are typically required to minimize the residual. Once the residual has been minimized, the concentration factor matrix is again determined by Eq. (34). Finally, the effective nonlinear response ${\overline{\mathbf{\Sigma }}}$ of a three-phase magnetoelectric composite is calculated via using Eq. (31).

At this point, one might assume the present three-phase unit-cell model is essentially the same as the traditional two-phase unit-cell model, as both of them utilize periodic parallelepipeds (subcells) to discretize a composite material. The fact is the three-phase model contains many unique features not found in the two-phase model. In Table 1 these differences are specifically identified in terms of 0–3 connectivity type. The aim of this table is to draw a clear distinction between the two- and three-phase models. For the purpose of comparison, we also include the Mori–Tanaka model, which will be presented shortly, in the Table 1. The intention here is not to undermine the well-established two-phase model and Mori–Tanaka approach or claim that they should be replaced, but rather to establish the present three-phase model as a method in its own right.

**Table 1:** Comparison between the present model and some micromechanics models in terms of 0–3 connectivity type.

Aspects	Three-phase unit-cell model (Present study)	Two-phase unit-cell model^31^	Mori–Tanaka model^41^
Geometric representation of a representative volume element (RVE)	A unit cell containing 64 parallelepiped subcells	A unit cell containing 8 parallelepiped subcells	A single ellipsoidal inclusion in an unbounded matrix
Maximum number of composite phases could be modeled	3	2	Unlimited
Capturing some variation of the inclusion fields	Yes (i.e., using 4 subcells to simulate the inclusion fields)	No (i.e., using 1 subcells to simulate the inclusion fields)	No
Capturing some variation of the matrix fields	Yes, it is a fine approximation owing to using 56 subcells to simulate the matrix fields	Yes, it however is a crude approximation due to using 7 subcells to simulate the matrix fields	No
Aspect ratio of the inclusion	Fixed ratio (i.e., only for 0–3, 1–3 and 2–2 composites^a^)	Fixed ratio (i.e., only for 0–3, 1–3 and 2–2 composites^a^)	Flexible ratio
Boundary conditions (BC)	Periodic BC	Periodic BC	Uniform BC

^a^In present study, 0–3 connectivity means that cubic particles are embedded in a matrix; 1–3 connectivity stands for that continuous fibers having square cross sections are embedded in a matrix; 2–2 connectivity represents that a bilaminated composite having rectangular cross sections for its two phases.

### Mori–Tanaka mean field approach

The Mori–Tanaka approach is commonly employed for the predictions of the effective moduli of two-phase smart composites comprising linear constituents only. Here, we reformulate this method for magnetoelectric composites insofar as nonlinear magnetostrictive and piezoelectric constituents are concerned and three-phase inclusion-matrix composites are addressed.

In a similar manner as for the average field of a unit cell in the simplified unit-cell micromechanics model, the average field of a representative volume element of a three-phase magnetoelectric composite is written as

36 $$ {\overline{\mathbf{Z}}} = \sum\limits_{r = 1}^{3} {c^{(r)} {\mathbf{Z}}^{(r)} } , $$

37 $$ {\overline{\mathbf{\Sigma }}} = \sum\limits_{r = 1}^{3} {c^{(r)} {{\varvec{\Sigma}}}^{(r)} } . $$

The superscript $(r)$ indicates the identity of a phase. For example, $r = 1$ is polymer matrix; $r = 2$ is magnetostrictive inclusion; $r = 3$ is piezoelectric reinforcement. $c^{(r)}$ is the volume fraction of the *r*th phase and satisfies the condition, $\sum\nolimits_{r = 1}^{3} {c^{(r)} } = 1$. The composite constitutive equation is later defined as (in an average sense)

38 $$ {\overline{\mathbf{\Sigma }}} = {\mathbf{\overline{L}\overline{Z}}}. $$

The phase constitutive equation is given by

39 $$ {{\varvec{\Sigma}}}^{(r)} = {\mathbf{L}}^{(r)} {\mathbf{Z}}^{(r)} . $$

Apply the concentration factor tensor ${\mathbf{A}}^{(r)}$^40^ to relate the field between the composite and its individual phase:

40 $$ {\mathbf{Z}}^{(r)} = {\mathbf{A}}^{(r)} {\overline{\mathbf{Z}}}. $$

Substitute Eq. (40) into Eq. (39) to replace ${\mathbf{Z}}^{(r)}$; then the resulting equation is used in Eq. (37) to replace ${{\varvec{\Sigma}}}^{(r)}$; as a result, the expression for the effective response is arrived below

41 $$ {\overline{\mathbf{\Sigma }}} = \sum\limits_{r = 1}^{3} {c^{(r)} {\mathbf{L}}^{(r)} {\mathbf{A}}^{(r)} } {\overline{\mathbf{Z}}}. $$

Compare Eq. (41) with Eq. (38); consequently the effective arithmetic stiffness of a three-phase magnetoelectric composite is obtained below

42 $$ {\overline{\mathbf{L}}} = \sum\limits_{r = 1}^{3} {c^{(r)} {\mathbf{L}}^{(r)} {\mathbf{A}}^{(r)} } . $$

Up to this point, the concentration factor tensor ${\mathbf{A}}^{(r)}$ is the only unknown to calculate the effective response ${\overline{\mathbf{\Sigma }}}$ and effective property ${\overline{\mathbf{L}}}$ in Eqs. (41) and (42), respectively. The Mori–Tanaka theory^41^ provides a unique way to determine the concentration factor tensor. It is the primary use of the elegant Eshelby^42^ formalism, based on eigenstrain concept, which is used to determine the solution to the problem of an ellipsoidal inclusion embedded in an infinite elastic matrix of material under uniform exterior mechanical loading. For a magnetoelectric material modeled by the Mori–Tanaka method, an inclusion is surrounded by the matrix with uniform strain and electric and magnetic fields the same as the matrix’s averaged strain and averaged electric and magnetic fields. The inclusion’s averaged fields are calculated from the solution for one ellipsoidal inclusion embedded in an infinite magneto-electro-elastic matrix. The extension from elastic problems to magneto-electro-elastic problems for three-phase composites makes the concentration factor tensor of the Mori–Tanaka approach having the following form:

43 $$ {\mathbf{A}}^{(r)} = {\mathbf{A}}^{dil,\;(r)} \left( {c^{(r)} {\mathbf{I}} + \sum\limits_{n = 2}^{3} {c^{(n)} {\mathbf{A}}^{dil,\;(n)} } } \right)^{ - 1} , $$

where ${\mathbf{A}}^{dil,\;(r)}$ is the concentration factor tensor of the so-called dilute scheme (or Eshelby approach) for the *r*th phase of a magnetoelectric composite, given as

44 $$ {\mathbf{A}}^{dil,\;(r)} = \left[ {{\mathbf{I}} + {\mathbf{S}}^{(r)} \left( {{\mathbf{L}}^{(1)} } \right)^{ - 1} \left( {{\mathbf{L}}^{(r)} - {\mathbf{L}}^{(1)} } \right)} \right]^{ - 1} . $$

${\mathbf{I}}$ in Eqs. (43) and (44) is a 12 × 12 identity matrix. ${\mathbf{S}}^{(r)}$ is the magneto-electro-elastic Eshelby tensor, expressed as

45 $$ S_{MnAb}^{(r)} = \left\{ \begin{gathered} \frac{1}{8\pi }L_{iJAb}^{(1)} \int_{ - 1}^{ + 1} {d\xi \int_{0}^{2\pi } {[G_{mJin} ({\mathbf{z}}) + G_{nJim} ({\mathbf{z}})]d\theta } } ,\quad M = 1,\;2,\;3, \hfill \\ \frac{1}{4\pi }L_{iJAb}^{(1)} \int_{ - 1}^{ + 1} {d\xi \int_{0}^{2\pi } {G_{4Jim} ({\mathbf{z}})d\theta } } ,\quad \quad \quad \quad \quad \;\,M = 4, \hfill \\ \frac{1}{4\pi }L_{iJAb}^{(1)} \int_{ - 1}^{ + 1} {d\xi } \int_{0}^{2\pi } {G_{5Jin} ({\mathbf{z}})} d\theta ,\quad \quad \quad \quad \quad \;\,\,M = 5, \hfill \\ \end{gathered} \right. $$

where

46 $$ G_{MJin} (z_{1} ,z_{2} ,z_{3} ) = K_{MJ}^{ - 1} z_{i} z_{n} , $$

47 $$ K_{JR} = z_{i} z_{n} L_{iJRn} , $$

48 $$ z_{1} = \frac{{y_{1} }}{{a_{1} }},\quad z_{2} = \frac{{y_{2} }}{{a_{2} }},\quad z_{3} = \frac{{y_{3} }}{{a_{3} }}, $$

49 $$ y_{1} = \sqrt {1 - \xi^{2} } \cos \theta ,\quad \quad y_{2} = \sqrt {1 - \xi^{2} } \sin \theta ,\quad \quad y_{3} = \xi . $$

A shorthand notation that treats the elastic, electric, and magnetic variables on an equal footing has adopted here. It is similar to conventional indicial notation with the exception that lowercase subscripts take on the range 1–3, while uppercase subscripts take on the range 1–5 and repeated uppercase subscripts are summed over 1–5. When an ellipsoidal inclusion is embedded in a general anisotropic magneto-electro-elastic material, ${\mathbf{S}}^{(r)}$ is given in terms of a double quadrature as shown in Eq. (45) that has to be carried out numerically. In the present study, however, the closed-form expressions of magneto-electro-elastic Eshelby tensor for the aligned elliptic-cylindrical inclusion (1–3 connectivity), spherical inclusion (0–3 connectivity), and thin-disc inclusion (2–2 connectivity) in an isotropic elastic medium are obtained, and are shown in Supplementary 2. In the case of linear constitutive laws applied for all phases in a magnetoelectric composite, Eq. (43) immediately gives a necessitate concentration factor tensor for Mori–Tanaka’s predictions. In contrast, when nonlinear constitutive laws are employed for magnetostrictive and piezoelectric phases in a multifunctional composite, the linearized constitutive relation in Eq. (24) would lead to an error estimation of the strain and electric and magnetic fields in each phase. This error can be defined by a residual as:

50 $$ \left\{ {{\mathbf{R}}^{{{\text{MT}}}} } \right\} = \left\{ \begin{gathered} {\mathbf{Z}}^{(1)} - {\mathbf{A}}^{(1)} {\overline{\mathbf{Z}}} \hfill \\ {\mathbf{Z}}^{(2)} - {\mathbf{A}}^{(2)} {\overline{\mathbf{Z}}} \hfill \\ {\mathbf{Z}}^{(3)} - {\mathbf{A}}^{(3)} {\overline{\mathbf{Z}}} \hfill \\ \end{gathered} \right\}. $$

After the residual is minimized, the Mori–Tanaka concentration factor tensor is evaluated by Eq. (43). Eventually, the effective nonlinear response ${\overline{\mathbf{\Sigma }}}$ of a three-phase magnetoelectric composite is obtained by the use of Eq. (41).

## Numerical implementation

This section is dedicated to the study of effective nonlinear responses in three-phase 1–3, 0–3, and 2–2 magnetoelectric composites under a prescribed set of magnetic fields. We first validate the simplified unit-cell model by comparing micromechanics predictions with limited experimental data available in literature. Next, we present parametric studies in order to reveal the effect of phase constitutive laws, phase volume fractions, and composite connectivities on the overall responses of magnetoelectric composites.

### Comparison with experimental data

**Figure 2:**
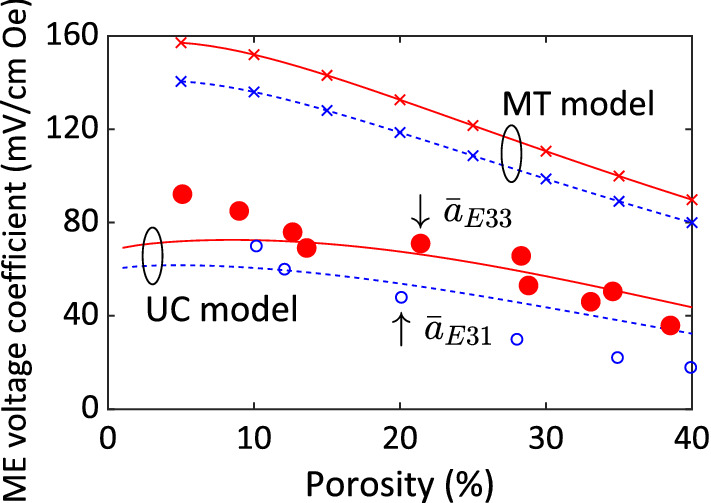
Comparison of micromechanical predictions and experimental measurements on the effective ME voltage coefficients: longitudinal coefficient $\overline{a}_{E33} = {{\overline{a}_{33}^{\varepsilon } } \mathord{\left/ {\vphantom {{\overline{a}_{33}^{\varepsilon } } {\overline{\kappa }_{33}^{\varepsilon ,H} }}} \right. \kern-\nulldelimiterspace} {\overline{\kappa }_{33}^{\varepsilon ,H} }}$(red solid lines and dots) and transverse coefficient $\overline{a}_{E31} = {{\overline{a}_{11}^{\varepsilon } } \mathord{\left/ {\vphantom {{\overline{a}_{11}^{\varepsilon } } {\overline{\kappa }_{33}^{\varepsilon ,H} }}} \right. \kern-\nulldelimiterspace} {\overline{\kappa }_{33}^{\varepsilon ,H} }}$(blue dashed lines and circles) for a fully constrained 60% nickel ferrite/40% PZT composite as a function of the percentage of porosity. The experimental data is obtained from Petrov et al.^43^. ME, MT and UC are abbreviations for magnetoelectric, Mori–Tanaka and unit-cell, respectively.

Most of experimental investigations for magnetoelectric composites are focused on two-phase composites. Three-phase setting for such composites is relatively limited in literature. However, few smart composites reported in literature can be still treated as three-phase magnetoelectric composites. For example, Petrov et al.^43^ experimentally examined the magnetoelectric effect in a porous magnetostrictive/piezoelectric bulk composite. The composite was made by PZT particles surrounded by a nickel ferrite matrix which had been modified to include porous microstructure. This composite can be regarded as a three-phase composite having PZT particles and voids in a nickel ferrite solid. The volume fraction of the PZT particles and the nickel ferrite matrix are 40% and 60%, respectively, where the 60% nickel ferrite matrix further shares some of spatial portion with the pores. The material properties required for micromechanics simulations are reported by Petrov et al.^43^ as well with the exception that instead of including nonlinear material parameters, the linear material coefficients were only used by them. The two micromechanics models with nonlinear responses developed in this study should be capable in predicting the overall linear magnetoelectric properties of magnetoelectric composites. In Fig. 2, it is seen that the unit-cell predictions (red solid line and blue dashed line) of the magnetoelectric voltage coefficients, $\overline{a}_{E33}$ and $\overline{a}_{E31}$ in longitudinal and transverse directions, respectively, are in good agreement with the experimental results (red dots and blue circles) for the pore volume fraction 0–40%. Although the Mori–Tanaka estimations (red solid-cross line and blue dashed-cross line) have the same trend as the unit-cell predictions but the Mori–Tanaka approximations show a relatively large deviation from the experimental results than the unit-cell model does. It may be owing to the various morphology used to approximate the PZT particles and pores. A cubic shape by the unit-cell model while a spherical geometry by the Mori–Tanaka scheme are utilized to approximate a particulate reinforcement. It also probably due to various number of fields employed to approximate the variation of the nickel ferrite matrix fields. Fifty-six matrix subcells enable the unit-cell model to capture some variation of the matrix fields while the Mori–Tanaka model discerns only the average matrix fields, i.e., a single value applicable to the entire matrix. It is however noted that the intention here is not to undermine the well-established Mori–Tanaka theory or claim that it should be replaced, but rather to establish the simplified unit-cell model as a method in its own right.

### Parametric studies

**Figure 3:**
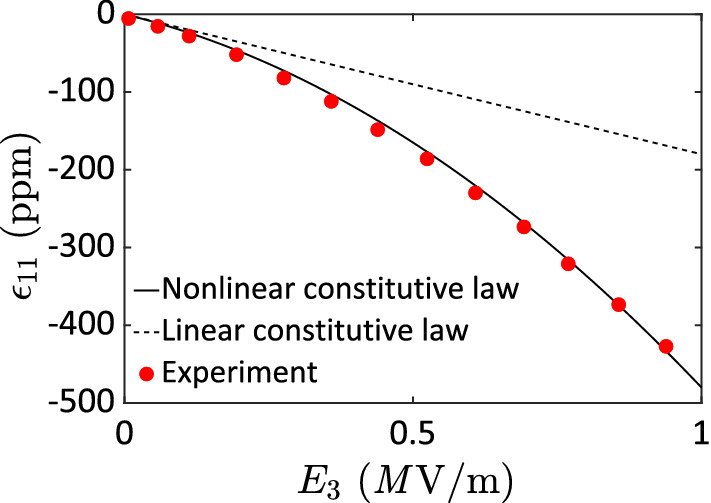
In-plate strain response $\varepsilon_{11}$ for a stress-free piezoelectric PZT-G1195 plate due to an applied electric field $E_{3}$ along the poling direction through the plate thickness (experimental data obtained from Crawley and Anderson^16^.

We conduct parametric studies here in order to investigate the overall nonlinear magnetoelectric responses particularly for a composite composed of PZT-G1195 and Terfenol-D that show significant material nonlinearity. Figure 3 depicts Crawley and Anderson’s^16^ experimental measurements (red dots) on the in-plate strain response $\varepsilon_{11}$ in a PZT-G1195 plate due to an applied electric field $E_{3}$ through the plate thickness aligned with the poling direction. It is obvious that the nonlinear constitutive law (solid line) can capture the entire range of the response while the linear constitutive equation (dashed line) works well only for low applied field, i.e., $E_{3} \approx$ 0.1 MV/m. As for engineering applications, it is desirable to utilize the greatest possible strain available in piezoelectric materials; as a result, a large electric driving field is needed, which results in significantly nonlinear piezoelectric responses.

**Figure 4:**
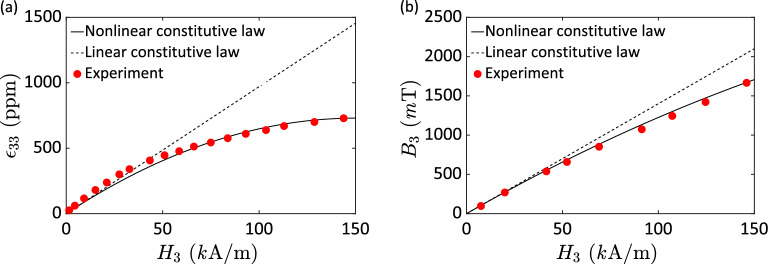
Strain (**a**) and magnetic flux density (**b**) responses of a stress-free magnetostrictive Terfenol-D rod due to an applied magnetic field (experimental data measured from a 20 kA/m bias field obtained from Jiles and Thoelke^17^.

Similarly, Fig. 4 illustrates Jiles and Thoelke’s^17^ experimental results (dots) on the strain $\varepsilon_{33}$ and magnetic flux density $B_{3}$ responses in a Terfenol-D rod owing to an applied magnetic field $H_{3}$. It is evident that the nonlinear constitutive relation (solid line) can predict the whole range of the responses while the linear constitutive law (dashed line) is useful for low magnetic field, i.e., $H_{3} \approx$ 30 *k*A/m. As for practical applications, it is desirable to utilize the greatest possible operating range for applied magnetic field available in magnetostrictive materials. In such case, pronouncedly nonlinear piezomagnetic responses are observed.

The nonlinear material parameters used to fit the experimental data of the PZT-G1195 and Terfenol-D specimens are listed in Table 2. For the subsequently parametric studies, an exemplary three-phase composite is taken as the nonlinear PZT-G1195 and Terfenol-D reinforcements in a linear elastic polymer matrix Araldite D. The material property of the Araldite D used for computation is shown in Table 2 as well.

**Table 2:** Material properties of constituents.

Property	Nickel ferrite^a^	PZT^a^	Terfenol-D^b^	PZT-G1195^c^	Araldite D^c^
$C_{1111}^{{}}$(10^9^ Pa)	494	142	8.541	63	8
$C_{1122}^{{}}$(10^9^ Pa)	381	92	0.654	34	4.4
$C_{1133}^{{}}$(10^9^ Pa)	417^f^	98	3.91	31	4.4
$C_{3333}^{{}}$(10^9^ Pa)	552^f^	139	28.3	49	8
$C_{2323}^{{}}$(10^9^ Pa)	56^f^	40^f^	5.55	22	1.8
$q_{311}^{{}}$(N/Am)	− 174.36	0	− 5.1056	0	0
$q_{333}^{{}}$(N/Am)	− 270.93	0	237.91	0	0
$q_{113}^{{}}$(N/Am)	28.09^f^	0	137.09	0	0
$e_{311}^{{}}$(C/m^2^)	0	− 1.89	0	− 6.3	0
$e_{333}^{{}}$(C/m^2^)	0	21.55	0	6.48	0
$e_{113}^{{}}$(C/m^2^)	0	23.72^f^	0	11.88	0
$\mu_{11}^{{}}$(10^−6^ N/A^2^)	3.76^f^	1.26^d^	30.914	6^e^	1.26^f^
$\mu_{33}^{{}}$(10^−6^ N/A^2^)	3.63	1.26^d^	11.644	11^e^	1.26^f^
$\kappa_{11}^{{}}$(10^−11^ F/m)	8.854^f^	126.56^f^	5^d^	863.69	3.5417
$\kappa_{33}^{{}}$(10^−11^ F/m)	8.854	621.58	5^d^	1045.10	3.5417
$x_{3311}^{{}}$(10^−5^ N/A^2^)	0	0	4.4344	0	0
$x_{3333}^{{}}$(10^−5^ N/A^2^)	0	0	− 157.32	0	0
$x_{1113}^{{}}$(10^−5^ N/A^2^)	0	0	− 53.724	0	0
$b_{3311}^{{}}$(10^−5^ F/m)	0	0	0	− 2.10	0
$b_{3333}^{{}}$(10^−5^ F/m)	0	0	0	2.16	0
$b_{1113}^{{}}$(10^−5^ F/m)	0	0	0	3.96	0
$\omega_{111}^{{}}$(10^−14^ m^2^ T/A^2^)	0	0	− 7253	0	0
$\omega_{333}^{{}}$(10^−14^ m^2^ T/A^2^)	0	0	− 1933	0	0
$\chi_{111}^{{}}$(10^−14^ F/V)	0	0	0	0	0
$\chi_{333}^{{}}$(10^−14^ F/V)	0	0	0	0	0

^a^The material properties of the nickel ferrite and PZT are obtained from Petrov et al.^43^.

^b^The material properties of the Terfenol-D are obtained from Shen and Lin^30^.

^c^The material properties of the PZT-G1195 and Araldite D are obtained from Lin and Muliana^31^.

^d^The corresponding material properties are obtained from Kuo and Peng^44^.

^e^The corresponding material properties are obtained from Veerannan and Arockiarajan^45^.

^f^ The corresponding material properties are assumed.

**Figure 5:**
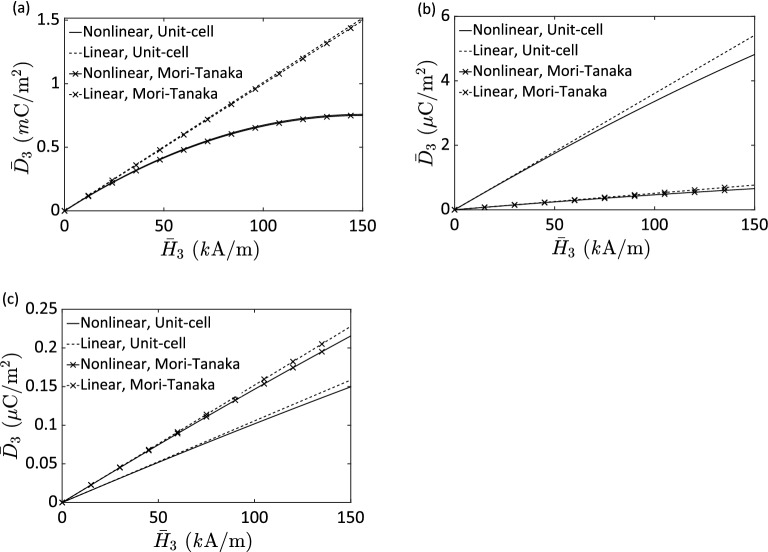
Effective electric displacement $\overline{D}_{3}$ as a function of an applied magnetic field $\overline{H}_{3}$ for a stress-free Terfenol-D/PZT G-1195/Araldite D composite with the corresponding phase volume fractions of 0.25/0.25/0.5 having 1–3 (**a**), 0–3 (**b**), and 2–2 (**c**) connectivities, respectively.

First of all, we investigate the nonlinear magnetoelectric responses in a three-phase composite having 1–3, 0–3 and 2–2 connectivities, respectively. The volume fractions of the PZT-G1195 and Terfenol-D inhomogeneities and the Araldite D matrix are taken of 0.25, 0.25 and 0.5, respectively. Both magnetization and electric polarization are aligned with the 3-direction that is the same as the fiber direction for 1–3 connectivity and is along with the layering direction for 2–2 type composite. Figure 5a shows the micromechanics predictions of the effective electric displacement $\overline{D}_{3}$ due to an applied magnetic field $\overline{H}_{3}$ for the 1–3 composite under a stress-free condition. Significant differences between nonlinear (solid lines) and linear (dashed lines) micromechanics estimations are observed. Besides, the unit-cell (without cross symbols) and Mori–Tanaka (with cross symbols) give close prediction regardless of linear or nonlinear cases in the 1–3 composite. The higher magnetic field is applied, the more pronounced nonlinearity is exhibited. It implies that it is necessary to consider the material nonlinearity when a three-phase magnetoelectric composite is subjected to a large applied field and is formed of the 1–3 connectivity, but it is not the case for 0–3 and 2–2 composites as shown in Fig. 5b,c. The magnetoelectric responses for the 0–3 and 2–2 composites show not only fewer overall responses but also less mismatch between linear and nonlinear estimations than 1–3 composite does. This is due to the fact that in the 0–3 and 2–2 magnetoelectric composites, the Araldite D polymer matrix, which is a passive constituent, dominates the overall responses; as a result, only a small fraction of the applied magnetic field reaches the magnetostrictive Terfenol-D reinforcements. It is also noted that the magnetic field $\overline{H}_{3}$ is applied along the fiber direction (3-direction), and the microstructural arrangement in the fiber composite allows for conducting this magnetic field directly through the Terfenol-D fibers, while in the case of the 0–3 and 2–2 composites, the inactive matrix limits the magnetic field in reaching the Terfenol-D particles and laminas, respectively. In addition, Fig. 5b depicts a relatively large discrepancy between the unit-cell and Mori–Tanaka results, regardless of linear or nonlinear cases, in the 0–3 composite in comparison with those in the 1–3 or 2–2 composites (Fig. 5a,c). It is because of the different geometries utilized in the unit-cell and Mori–Tanaka models to simulate the particle reinforcements in the 0–3 composite as discussed previously. This combination of findings supplies some support for the conceptual premise that nonlinear active constituents lead to significant overall nonlinear responses of three-phase magnetoelectric composites when the composites are subjected to large applied fields. Moreover, a three-phase magnetoelectric composite with continuous active fibers provides the best performance.

**Figure 6:**
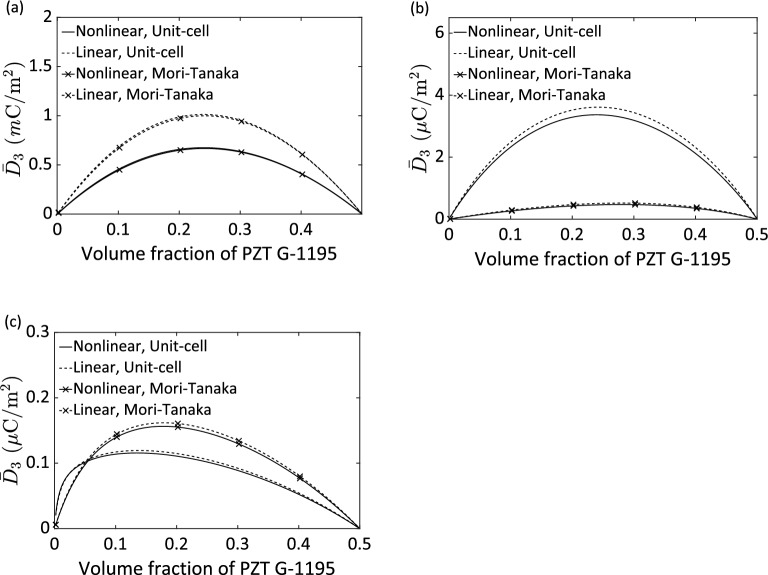
Effective electric displacement $\overline{D}_{3}$ as a function of PZT G-1195 volume fraction for a stress-free Terfenol-D/PZT G-1195/Araldite D composite subjected to a constant magnetic field $\overline{H}_{3}$ = 100 *k*A/m having 1–3 (**a**), 0–3 (**b**), and 2–2 (**c**) connectivities, respectively. The volume fraction of the Araldite D is fixed of 0.5, while the Terfenol-D and PZT G-1195 phases share the rest of the volume fraction, namely, 0.5.

Second, we examine the effect of the volume fraction of the active constituents, i.e., PZT-G1195 and Terfenol-D, on the overall responses of a three-phase composite. We keep the volume fraction of the Araldite D matrix as a constant value of 0.5 and then vary the volume fraction of the PZT-G1195 reinforcement from 0 to 0.5 against the one of the Terfenol-D inhomogeneity from 0.5 to 0. All settings of the connectivity and the direction of magnetization, electric polarization, fiber, and layering are the same as the previous parametric study except for the active phase volume fraction. Figure 6 illustrates the effective electric displacement $\overline{D}_{3}$ as a function of the volume fraction of the PZT-G1195 for a stress-free magnetoelectric 1–3, 0–3 and 2–2 composite, respectively, subjected to a constant magnetic field, i.e., $\overline{H}_{3}$ = 100 *k*A/m. It is of course that the magnetoelectric coupling vanishes when only one active phase (PZT-G1195 or Terfenol-D) is present; that is shown by the zero response of $\overline{D}_{3}$ at the PZT-G1195 volume fraction of 0 or 0.5. In contrast, a relatively large magnetoelectric coupling can be obtained by evening the proportion of two active phase. In the present example, it can be seen at the PZT-G1195 volume fraction of around 0.25. This is due to that the magnetoelectric coupling is engineered by the two active phases, PZT-G1195 and Terfenol-D, in a three-phase composite. Evened the proportion of the two active phases maximizes the overall magnetoelectric coupling. As the previous finding in the first parametric study, the unit-cell and Mori–Tanaka predictions are close to each other for the 1–3 composite (Fig. 6a) regardless of linear or nonlinear cases. On the other hand, for the 0–3 and 2–2 types (Fig. 6b,c) the difference between the linear and nonlinear predictions is insignificant regardless of the unit-cell or Mori–Tanka predictions, although the two micromechanics estimations are deviated from each other. It is probably due to, as the foregoing discussion, only a small amount of the magnetic field reaches the magnetostrictive Terfenol-D particles and layers, respectively. These findings deduce that it is necessary to employ the nonlinear constitutive laws for the design of a three-phase magnetoelectric composite having 1–3 connectivity. These findings also raise an intriguing question regarding the prediction accuracy of the two micromechanics models applying to the cases of the 0–3 and 2–2 connectivities. The accuracy is required to be evaluated by a large amount of experimental data, which is outside the scope of this study.

**Figure 7:**
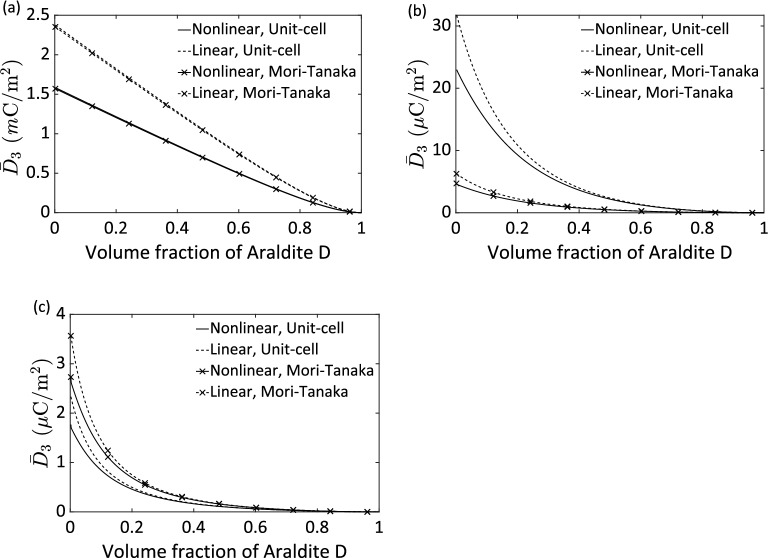
Effective electric displacement $\overline{D}_{3}$ as a function of Araldite D volume fraction for a stress-free Terfenol-D/PZT G-1195/Araldite D composite subjected to a constant magnetic field $\overline{H}_{3}$ = 100 *k*A/m having 1–3 (**a**), 0–3 (**b**), and 2–2 (**c**) connectivities, respectively. The ratio of the volume fraction of the Terfenol-D to the PZT G-1195 is kept as 1:1.

Finally, we explore the effect of the proportion of the passive phase, i.e., Araldite D, on the effective responses of a three-phase composite. All prescribed conditions for this simulation are the same as the last parametric study. At this moment, however, we retain a constant ratio of the volume fractions of the two active phases to be 1:1, and then modulate the volume fraction of the Araldite D matrix from 0 to 1. Figure 7 shows the effective response of electric displacement $\overline{D}_{3}$ as a function of the volume fraction of the passive Araldite D matrix for the three types of connectivity, respectively. It is expected that the magnetoelectric coupling reaches the extrema at the Araldite D volume fraction of either 0 or 1, respectively. Between them, the magnetoelectric coupling descends as the proportion of the Araldite D ascends. It suggests that the maximum magnetoelectric effect can be achieved when the Araldite D matrix vanishes. However, the exclusion of the polymer matrix from a three-phase composite, it may cause a noncompliant composite that is in general not preferable for practical applications. In other words, a flexible polymer matrix makes a three-phase magnetoelectric composite more compliant at the expense of reducing overall magnetoelectric coupling. It further deduces that the present micromechanics models provide a means to reveal the overall nonlinear response, which is quite essential for tailoring a three-phase magnetoelectric composite for desirable performance. An important physical mechanism is also unveiled by all the above numerical results. The magnetoelectric effect in a three-phase composite is caused by mechanically coupled magnetostrictive and piezoelectric phases that are both surrounded by a polymer matrix: it is present in none of the individual phase separately. Under magnetic field owing to the piezomagnetism of the magnetostrictive constituent, there are mechanical strains which are elastically transmitted through the polymer intermediator onto the piezoelectric phase resulting in polarization changes via piezoelectricity.

## Conclusion

Three-phase nonlinear magnetoelectric composites have been studied using the simplified unit-cell model. In development of the mathematical foundation, we utilize concepts from conventional micromechanics discipline such as representative volume elements, volume‐weighted averages, and concentration factor tensors. The significance of this formulation is that a first-moment secant linearization is employed and is then nested into the micromechanics model followed by an iterative scheme in order to minimize the error resulted from the linearization process. The outcome of the simplified unit-cell model is the predations of effective nonlinear responses and its results are comparable to those from the well-established Mori–Tanaka model. We elucidate the similarity between the two micromechanics models in terms of not only their mathematical structures but also their numerical results. The developed unit-cell model can serve as a constitutive equation of a three-phase magnetoelectric composite material, that can be further integrated into finite element processes, via integration points, to modeling of composite structures such as beams, plates, and shells, which is a great merit in a variety of practical engineering applications.

The main points found from the numerical implementation are summarized here. First, the developed simplified unit-cell model with 64 subcells has validated against experimental data. Second, the overall responses of a three-phase magnetoelectric composite is significantly influenced by nonlinear constituents particularly when the composite is subjected to large applied fields. Third, 1–3 connectivity provides the best figure of merit than the magnetoelectric composites have 0–3 or 2–2 connectivities. Fourth, including a polymer matrix lets a magnetoelectric composite to be easily shaped but would also deteriorate the overall magnetoelectric coupled performance. Fifth, the micromechanics predictions from the simplified unit-cell and Mori–Tanaka models are in general close to each other especially for 1–3 magnetoelectric composites. Taken together, the contributions of this work complement those of earlier studies that prevailingly focused on two-phase composites and linear constitutive responses, in general.

## Supplementary Information

Supplementary Information.

## Data Availability

The datasets generated during or analysed during the current study are available from the corresponding author on reasonable request.
