# Supplementary material for: Effective nonlinear responses of three-phase magnetoelectric composites
Source: Sci Rep. 2022 Sep 6;12:15101. doi: 10.1038/s41598-022-19143-2 (PMC9448742; doi:10.1038/s41598-022-19143-2)
Supplement: Supplementary file 1 — Supplementary Information. [file 41598_2022_19143_MOESM1_ESM.docx]

Effective Nonlinear Responses of Three-phase Magnetoelectric Composites

Chien-hong Lin* and Fang-Yu Liu

Department of Mechanical Engineering, National Cheng Kung University, 1 University Road, Tainan City 70101, Taiwan
*Corresponding author: [clin@gs.ncku.edu.tw](mailto:clin@gs.ncku.edu.tw), phone 06-2757575 ext. 62139

**Supplementary 1**

This supplementary lists the micromechanical relations of the simplified unit‐cell model having 64 subcells as illustrated in Fig. S1. The homogenized strain equations are derived by imposing the periodic continuity conditions of displacements, and are summarized below.

$\bar{\varepsilon}_{11}=\frac{V^{(i)}\varepsilon_{11}^{\text{(}i\text{)}}+V^{(i+1)}\varepsilon_{11}^{\text{(}i+1\text{)}}+V^{(i+2)}\varepsilon_{11}^{\text{(}i+2\text{)}}+V^{(i+3)}\varepsilon_{11}^{\text{(}i+3\text{)}}}{V^{(i)}+V^{(i+1)}+V^{(i+2)}+V^{(i+3)}}, \begin{matrix} i=1,5,9,13,17,21,25,29,33, \\ 37,41,45,49,53,57,61, \end{matrix}$ (A1)

$\bar{\varepsilon}_{22}=\frac{V^{(i)}\varepsilon_{22}^{\text{(}i\text{)}}+V^{(i+4)}\varepsilon_{22}^{\text{(}i+4\text{)}}+V^{(i+8)}\varepsilon_{22}^{\text{(}i+8\text{)}}+V^{(i+12)}\varepsilon_{22}^{\text{(}i+12\text{)}}}{V^{(i)}+V^{(i+4)}+V^{(i+8)}+V^{(i+12)}}, \begin{matrix} i=1,2,3,4,17,18,19,20,33, \\ 34,35,36,49,50,51,52, \end{matrix}$ (A2)

$\bar{\varepsilon}_{33}=\frac{V^{(i)}\varepsilon_{33}^{\text{(}i\text{)}}+V^{(i+16)}\varepsilon_{33}^{\text{(}i+16\text{)}}+V^{(i+32)}\varepsilon_{33}^{\text{(}i+32\text{)}}+V^{(i+48)}\varepsilon_{33}^{\text{(}i+48\text{)}}}{V^{(i)}+V^{(i+16)}+V^{(i+32)}+V^{(i+48)}}, i=1..16.$ (A3)

$2\bar{\varepsilon}_{23}=\frac{\sum_{j} V^{(j+k)}2\varepsilon_{23}^{(j+k)}}{\sum_{i} V^{(i+k)}}, \begin{matrix} i=j=1,5,9,13,17,21,25,29,33,37,41,45,49,53,57,61, \\ k=0,1,2,3, \end{matrix}$ (A4)

$2\bar{\varepsilon}_{13}=\frac{\sum_{j} V^{(j+k)}2\varepsilon_{13}^{(j+k)}}{\sum_{i} V^{(i+k)}}, \begin{matrix} i=j=1,2,3,4,17,18,19,20,33,34,35,36,49,50,51,52, \\ k=0,4,8,12, \end{matrix}$ (A5)

$2\bar{\varepsilon}_{12}=\frac{\sum_{j} V^{(j+k)}2\varepsilon_{12}^{(j+k)}}{\sum_{i} V^{(i+k)}}, \begin{matrix} i=j=1..16, \\ k=0,16,32,48, \end{matrix}$ (A6)

The stress equations are derived by imposing the continuity conditions of tractions at the interfaces of the subcells, and are summarized below.$\sigma_{11}^{(i)}=\sigma_{11}^{(i+1)}=\sigma_{11}^{(i+2)}=\sigma_{11}^{(i+3)}, i=1,5,9,13,17,21,25,29,33,37,41,45,49,53,57,61,$ (A7)

$\sigma_{22}^{(i)}=\sigma_{22}^{(i+4)}=\sigma_{22}^{(i+8)}=\sigma_{22}^{(i+12)}, i=1,2,3,4,17,18,19,20,33,34,35,36,49,50,51,52,$ (A8)

$\sigma_{33}^{(i)}=\sigma_{33}^{(i+16)}=\sigma_{33}^{(i+32)}=\sigma_{33}^{(i+48)}, i=1..16.$ (A9)

$\sigma_{23}^{(i)}=\sigma_{23}^{(i+j)}, \begin{matrix} i=1,2,3,4, \\ j=4,8,12,16,20,24,28,32,36,40,44,48,52,56,60, \end{matrix}$ (A10)

$\sigma_{13}^{(i)}=\sigma_{13}^{(i+j)}, \begin{matrix} i=1,5,9,13, \\ j=1,2,3,16,17,18,19,32,33,34,35,48,49,50,51, \end{matrix}$ (A11)

$\sigma_{12}^{(i)}=\sigma_{12}^{(i+j)}, \begin{matrix} i=1,17,33,49, \\ j=1..15, \end{matrix}$ (A12)

The homogenized electric field equations listed below are deduced by using the periodic continuity conditions of electric potentials.

$\bar{E}_{1}=\frac{V^{(i)}E_{1}^{\text{(}i\text{)}}+V^{(i+1)}E_{1}^{\text{(}i+1\text{)}}+V^{(i+2)}E_{1}^{\text{(}i+2\text{)}}+V^{(i+3)}E_{1}^{\text{(}i+3\text{)}}}{V^{(i)}+V^{(i+1)}+V^{(i+2)}+V^{(i+3)}}, \begin{matrix} i=1,5,9,13,17,21,25,29,33, \\ 37,41,45,49,53,57,61, \end{matrix}$ (A13)

$\bar{E}_{2}=\frac{V^{(i)}E_{2}^{\text{(}i\text{)}}+V^{(i+4)}E_{2}^{\text{(}i+4\text{)}}+V^{(i+8)}E_{2}^{\text{(}i+8\text{)}}+V^{(i+12)}E_{2}^{\text{(}i+12\text{)}}}{V^{(i)}+V^{(i+4)}+V^{(i+8)}+V^{(i+12)}}, \begin{matrix} i=1,2,3,4,17,18,19,20,33, \\ 34,35,36,49,50,51,52, \end{matrix}$ (A14)

$\bar{E}_{3}=\frac{V^{(i)}E_{3}^{\text{(}i\text{)}}+V^{(i+16)}E_{3}^{\text{(}i+16\text{)}}+V^{(i+32)}E_{3}^{\text{(}i+32\text{)}}+V^{(i+48)}E_{3}^{\text{(}i+48\text{)}}}{V^{(i)}+V^{(i+16)}+V^{(i+32)}+V^{(i+48)}}, i=1..16.$ (A15)

The equations for electric displacement are derived by imposing the continuity conditions of normal electric displacements at the interfaces among the subcells, and are summarized below.

$D_{1}^{(i)}=D_{1}^{(i+1)}=D_{1}^{(i+2)}=D_{1}^{(i+3)}, i=1,5,9,13,17,21,25,29,33,37,41,45,49,53,57,61,$ (A16)

$D_{2}^{(i)}=D_{2}^{(i+4)}=D_{2}^{(i+8)}=D_{2}^{(i+12)}, i=1,2,3,4,17,18,19,20,33,34,35,36,49,50,51,52,$ (A17)

$D_{3}^{(i)}=D_{3}^{(i+16)}=D_{3}^{(i+32)}=D_{3}^{(i+48)}, i=1..16.$ (A18)

The homogenized magnetic field equations are derived by employing the periodic continuity conditions of magnetic potentials, and are shown below.

$\bar{H}_{1}=\frac{V^{(i)}H_{1}^{\text{(}i\text{)}}+V^{(i+1)}H_{1}^{\text{(}i+1\text{)}}+V^{(i+2)}H_{1}^{\text{(}i+2\text{)}}+V^{(i+3)}H_{1}^{\text{(}i+3\text{)}}}{V^{(i)}+V^{(i+1)}+V^{(i+2)}+V^{(i+3)}}, \begin{matrix} i=1,5,9,13,17,21,25,29,33, \\ 37,41,45,49,53,57,61, \end{matrix}$ (A19)

$\bar{H}_{2}=\frac{V^{(i)}H_{2}^{\text{(}i\text{)}}+V^{(i+4)}H_{2}^{\text{(}i+4\text{)}}+V^{(i+8)}H_{2}^{\text{(}i+8\text{)}}+V^{(i+12)}H_{2}^{\text{(}i+12\text{)}}}{V^{(i)}+V^{(i+4)}+V^{(i+8)}+V^{(i+12)}}, \begin{matrix} i=1,2,3,4,17,18,19,20,33, \\ 34,35,36,49,50,51,52, \end{matrix}$ (A20)

$\bar{H}_{3}=\frac{V^{(i)}H_{3}^{\text{(}i\text{)}}+V^{(i+16)}H_{3}^{\text{(}i+16\text{)}}+V^{(i+32)}H_{3}^{\text{(}i+32\text{)}}+V^{(i+48)}H_{3}^{\text{(}i+48\text{)}}}{V^{(i)}+V^{(i+16)}+V^{(i+32)}+V^{(i+48)}}, i=1..16.$ (A21)

The equations for magnetic flux density are derived by imposing the continuity conditions of normal magnetic flux densities at the interfaces among the subcells, and are summarized below.

$B_{1}^{(i)}=B_{1}^{(i+1)}=B_{1}^{(i+2)}=B_{1}^{(i+3)}, i=1,5,9,13,17,21,25,29,33,37,41,45,49,53,57,61,$ (A22)

$B_{2}^{(i)}=B_{2}^{(i+4)}=B_{2}^{(i+8)}=B_{2}^{(i+12)}, i=1,2,3,4,17,18,19,20,33,34,35,36,49,50,51,52,$ (A23)

$B_{3}^{(i)}=B_{3}^{(i+16)}=B_{3}^{(i+32)}=B_{3}^{(i+48)}, i=1..16.$ (A24)

**49 50 51 52**

**53 54 55 56**

**57 58 59 60**

**61 62 63 64**

Back layer

**33 34 35 36**

**37 38 39 40**

**41 42 43 44**

**45 46 47 48**

*x*_1_

*x*_2_

*x*_3_

Three-phase 0-3 ME composite

**17 18 19 20**

**21 22 23 24**

**25 26 27 28**

**29 30 31 32**

Idealized 0-3 ME composite

**1 2 3 4**

**5 6 7 8**

**9 10 11 12**

**13 14 15 16**

**1 2 3 4**

**5 6 7 8**

**9 10 11 12**

**13 14 15 16**

**64**

Homogenized comparison material

Front layer

Unit cell

Figure S1. Homogenization process of the simplified unit-cell micromechanics model for a three-phase 0-3 ME composite. ME an abbreviation for magnetoelectric.

Furthermore, not only for 0-3 connectivity as shown in Fig. S1. but also for 1-3 and 2-2 connectivities, the unit cell having 64 subcells is able to address them as well. For 1-3 type, as shown in Fig. S2, magnetostrictive fibers locate on the subcell 1, 17, 33, 49, 11, 27, 43, and 59; piezoelectric fibers occupy the subcell 3, 19, 35, 51, 9, 25, 41, and 57; polymer matrix uses the rest of the subcells. For 2-2 connectivity, as depicted in Fig. S3, magnetostrictive layers locate on the subcell 1, 2, 3, 4, 17, 18, 19, 20, 33, 34, 35, 36, 49, 50, 51, and 52; piezoelectric layers occupy the subcell 9, 10, 11, 12, 25, 26, 27, 28, 41, 42, 43, 44, 57, 58, 59, and 60; polymer matrix utilizes the rest of the subcells. In short, the simplified unit-cell model with 64 subcells provides flexibility to simulate a magnetoelectric composite either having three phases or having three basic connectivities.

**49 50 51 52**

**53 54 55 56**

**57 58 59 60**

**61 62 63 64**

Back layer

**33 34 35 36**

**37 38 39 40**

**41 42 43 44**

**45 46 47 48**

*x*_1_

*x*_2_

*x*_3_

Three-phase 1-3 ME composite

**17 18 19 20**

**21 22 23 24**

**25 26 27 28**

**29 30 31 32**

Idealized 1-3 ME composite

**1 2 3 4**

**5 6 7 8**

**9 10 11 12**

**13 14 15 16**

**1 2 3 4**

**5 6 7 8**

**9 10 11 12**

**13 14 15 16**

**64**

Homogenized comparison material

Front layer

Unit cell

Figure S2. Homogenization process of the simplified unit-cell micromechanics model for a three-phase 1-3 ME composite.

**49 50 51 52**

**53 54 55 56**

**57 58 59 60**

**61 62 63 64**

Back layer

**33 34 35 36**

**37 38 39 40**

**41 42 43 44**

**45 46 47 48**

*x*_1_

*x*_2_

*x*_3_

Three-phase 2-2 ME composite

**17 18 19 20**

**21 22 23 24**

**25 26 27 28**

**29 30 31 32**

Idealized 2-2 ME composite

**1 2 3 4**

**5 6 7 8**

**9 10 11 12**

**13 14 15 16**

**1 2 3 4**

**5 6 7 8**

**9 10 11 12**

**13 14 15 16**

**64**

Homogenized comparison material

Front layer

Unit cell

Figure S3. Homogenization process of the simplified unit-cell micromechanics model for a three-phase 2-2 ME composite.

**Supplementary 2**

This supplementary lists the components of the magneto-electro-elastic Eshelby tensor for three different geometries of inclusions, respectively. First, for an infinitely long cylindrical inclusion of circular cross section in an isotropic matrix with Poisson's ratio $v$, it follows from Eq. that in the limit $a_{1}$=$a_{2}$=1 and $a_{3}\to\infty$, the nonzero components of the magneto-electro-elastic Eshelby tensor thus given below.

$$S_{1111}=S_{2222}=\frac{4v-5}{8v-8},$$

$$S_{1122}=S_{2211}=\frac{1-4v}{8v-8},$$

$$S_{1133}=S_{2233}=\frac{v}{2-2v},$$

$$S_{1212}=S_{2112}=S_{1221}=S_{2121}=\frac{4v-3}{8v-8},$$

$$S_{2323}=S_{3223}=S_{2332}=S_{3232}=S_{3131}=S_{1331}=S_{3113}=S_{1313}=\frac{1}{4},$$

$$S_{1414}=S_{4114}=S_{1441}=S_{4141}=S_{2424}=S_{4224}=S_{2442}=S_{4242}=\frac{1}{2},$$

$$S_{1515}=S_{5115}=S_{1551}=S_{5151}=S_{2525}=S_{5225}=S_{2552}=S_{5252}=\frac{1}{2}.$$

Second, for a spherical inclusion that in the range $a_{1}$=$a_{2}$=$a_{3}$=1 in an isotropic matrix, the nonzero components of the magneto-electro-elastic Eshelby tensor are summarized here.

$$S_{1111}=S_{2222}=S_{3333}=\frac{5v-7}{15v-15},$$

$$S_{1122}=S_{2211}=S_{1133}=S_{3311}=S_{2233}=S_{3322}=\frac{1-5v}{15v-15},$$

$$S_{1122}=S_{2211}=S_{1133}=S_{3311}=S_{2233}=S_{3322}=\frac{1-5v}{15v-15},$$

$$S_{2323}=S_{3223}=S_{2332}=S_{3232}=S_{3131}=S_{1331}=S_{3113}=S_{1313}=S_{1212}=S_{2112}=S_{1221}=S_{2121}=\frac{5v-4}{15v-15},$$

$$S_{1414}=S_{4114}=S_{1441}=S_{4141}=S_{2424}=S_{4224}=S_{2442}=S_{4242}=S_{3434}=S_{4334}=S_{3443}=S_{4343}=\frac{1}{3},$$

$$S_{1515}=S_{5115}=S_{1551}=S_{5151}=S_{2525}=S_{5225}=S_{2552}=S_{5252}=S_{3535}=S_{5335}=S_{3553}=S_{5353}=\frac{1}{3}.$$

Third, for a layered inclusion that in the setting, $a_{1}=a_{2}\to\infty$and $a_{3}$=1, in an isotropic matrix, the nonzero components of the magneto-electro-elastic Eshelby tensor are enumerated as follows.

$$S_{3311}=S_{3322}=\frac{v}{1-v},$$

$$S_{3333}=1,$$

$$S_{2323}=S_{3223}=S_{2332}=S_{3232}=S_{3131}=S_{1331}=S_{3113}=S_{1313}=\frac{1}{2},$$

$$S_{3434}=S_{4334}=S_{3443}=S_{4343}=1,$$

$$S_{3535}=S_{5335}=S_{3553}=S_{5353}=1.$$
